# ECPR for prolonged Pediatric Cardiac Arrest, an outcome without major neurological compromise

**DOI:** 10.1051/ject/2023019

**Published:** 2023-12-15

**Authors:** Laura Gutiérrez-Soriano, Eduardo Becerra Zapata, Nicolas Maya Trujillo, German Andres Franco Gruntorad, Pedro Hurtado Peña

**Affiliations:** 1 Cardiovascular Anesthesiologist Anesthesiology Department, Fundación Cardioinfantil 1113111 Bogota Colombia; 2 Anesthesiology Department, Fundación Cardioinfantil 1113111 Bogota Colombia; 3 Anesthesiology Department, Hospital Universitario San Ignacio 1113111 Bogota Colombia

**Keywords:** Pediatrics extracorporeal membrane oxygenation, Cardiac arrest, Extracorporeal cardiopulmonary resuscitation, Ischemic preconditioning, Congenital heart disease, Postoperative care

## Abstract

Pediatric in-hospital cardiac arrest (IHCA) has been reported in 1–3% of pediatric intensive care unit (ICU) admissions and up to 6% of children admissions to the cardiac ICU. In the last 25 years, the survival to hospital discharge after pediatric IHCA has improved from 9% to 13.7% up to 35%. The improvement in outcomes was attributed in part to the application of ECMO as a rescue strategy when prolonged conventional CPR cannot restore spontaneous circulation. We report a case of a 4-month-old patient with a history of ventricular and septal defects, with left to right shunt and enlargement of left heart chambers that underwent surgery for the closure of the atrial and septal defects, and experienced complications that led to the use of ECMO in response to a prolonged cardiac arrest.

## Introduction

This report describes the surgical procedure of a 4-month-old boy with ventricular septum and atrial septal defects who underwent a medium sternotomy. The surgical closure of the ventricular septal defect and primary surgical closure of the atrial septal defect were performed without complications. However, the patient experienced oxygen desaturation, bradycardia, and asystole in the early perioperative period while respiratory therapy was performed, leading to a cardiac arrest. Emergent sternotomy and open heart massage were performed, and the patient was put on VA ECMO (Venoarterial Extracorporeal Membrane Oxygenation) support after 1 h of cardiac arrest. Despite the initial suboptimal flow given by the patient’s hemodynamic instability with VA ECMO support, the patient eventually recovered and underwent decannulation and repair of the residual ventricular septal defect under cardiopulmonary bypass. The patient had a successful recovery but showed mild abnormally low muscle tone at discharge. This case report highlights the successful management of a pediatric patient who experienced a cardiac arrest during the perioperative period with the use of venoarterial ECMO support.

**Abbreviations**
E-CPR: Extracorporeal Cardiopulmonary Resuscitation
ECMO: Once in ECMO
CNN: During Cannulation
CPR: Cardiopulmonary Resuscitation
ICU: Intensive Care Unit
IHCA: In-Hospital Cardiac Arrest
IPP: Immediate Postoperative Period
Pre Sx: Pre Surgical
VA ECMO: VenoArterial Extracorporeal Membrane Oxygenation

## Case presentation

Here we discuss a 4-month-old male patient, with a history of ventricular septum defect of 11 mm and atrial septal defect of 5 mm with left to right shunt and a mild enlargement of the left ventricle and atria, without pulmonary hypertension and normal biventricular function. The patient underwent surgery through a medium sternotomy. After aortic and bicaval cannulation, an aortic cross-clamp was performed, antegrade del Nido cardioplegia was achieved with 80 mL of plasmalyte solution with a single dose of 43 s. The Ventricular septal defect was closed with a Gore-Tex patch (G. Barco S.A, Bogota Colombia) and primary surgical closure was performed to the atrial septal defect. The total perfusion and aortic clamp time was 76 min and 43 min respectively. The patient was weaned from bypass without complications and was transferred to the intensive care unit with mechanical ventilation and a norepinephrine dose of 0.02 mcg/kg/min, and laboratory results as seen in Table 1 in the immediate postoperative period.

**Table 1:** Laboratory tests progression. Pre Sx: Presurgical; IPP: Immediate postoperative period; CNN: During cannulation; ECMO: Once in ECMO.

Lab test	Pre Sx	IPP	CNN	ECMO		Day 1	Day 2	Day 3	Day 4
Hemoglobin (g/dL)	10.81	10	12	9	9.5	13	11	12	11.3
Hematocrit (%)	33	29	33	22	25	42	32	36	31.4
pH	7.46	7.53	7.17	7.19	6.7	7.13	7.37	7.4	7.3
pCO_2_ (mmHg)	18.7	8.7	7.4	13	58	58	33	33	58.5
pO_2_ (mmHg)	30.9	52	190	241	67	156	185	211	77.8
HCO_3_ (mEq/L)	21.8	13	6.7	8.2	7.8	21	19	21	29.1
BE (mEq/L)	−1.2	15	−22	−20	−27	−6	−5.4	−3.7	1.9
SATO_2_ (%)	99.3	94	99	99	64	98	99	99	99
FiO_2_ (%)	50	100	100	100	100	100	100	100	80
Lactate (mg/dL)	1	7.26	17	13.6	11	2.3	1.5	1.4	1.9

Oxygen desaturation, bradycardia, and asystole were documented in the early perioperative period while the patient was in a respiratory therapy session. Immediately, high-quality CPR (cardiopulmonary resuscitation) was initiated and echocardiographic findings showed severe biventricular enlargement with akinesia of the right ventricle and hypokinesia of the left ventricle. Emergent sternotomy and open heart massage were performed achieving arterial blood pressures around 90/50 mmHg and 100/60 mmHg. Because of edema and heart enlargement, central cannulation for VA ECMO (venoarterial ECMO) was done after 1 h of cardiac arrest.

After VA ECMO was initiated, suboptimal flow was reached despite venous cannula manipulation and transfusion of RBC, (red blood cells) plasma and albumin administration. Anisocoria with nonreactive pupils was documented. After our inability to flow on ECMO as well as a very high lactate level, the decision was made to stop ECMO and return to CPR (Table 1). After the venous cannula was removed and reinserted, optimal flow with the ECMO was reached and a new arterial blood sample was taken, showing a lactate of 13. ECMO was resumed after 1 h and 30 min of cardiac arrest and lasted for 2 days. A neurological examination was performed.

The patient’s anisocoria resolved within 24 h of V-A ECMO support and lactate levels also decreased to normal (Table 1). An LV Vent was added to the left ventricle because echocardiographic findings showed severe biventricular enlargement and dysfunction. Despite optimal anticoagulation, thrombosis of the vent was documented on the second day of support, requiring vent exchange. Transfontanel ultrasound was performed and shown to be normal, and on the third brain, tomography was also normal. A transthoracic Cardiac Echo showed biventricular function improving with an LVEF (left ventricular ejection fraction) of 40% and two residual ventricular septal defects. Patient underwent decannulation and repair of the residual ventricular septal defect under cardiopulmonary bypass, with 60 min of aortic cross-clamp and 110 min of perfusion.

During the early post-operative ICU stay the patient was treated with milrinone, norepinephrine, and vasopressin infusions. ECMO therapy had a duration of two days, mechanical ventilatory weaning was done on day 24 and extubation was performed without complications. The patient was transferred to a general ward and then discharged home after 40 days. Mild abnormally low muscle tone was observed at discharge, without other neurological deficits, and with proper extremity movement.

## Discussion

Pediatric in-hospital cardiac arrest (IHCA) has been reported in 1–3% of pediatric intensive care units (ICU) admissions and up to 6% of children admitted to a cardiac ICU [1]. In the last 25 years, the survival to hospital discharge after pediatric IHCA has improved from 9% to 13.7% and up to 35% [2, 3]. The improvement in outcomes was attributed partly to the impact of ECMO as a rescue strategy when prolonged conventional CPR cannot restore spontaneous circulation [1]. As shown by results from the large GWTG-R IHCA database, which included pediatric patients treated with at least 10 min of in-hospital CPR, showing that patients who received ECPR had higher odds of surviving to discharge than those who received prolonged conventional CPR [4].

In ECPR (Extracorporeal Cardiopulmonary Resuscitation) patients with sudden or unexpected pulselessness caused by the cessation of cardiac mechanical activity receive veno-arterial extracorporeal membrane oxygenation (VA-ECMO). Extracorporeal life support organization (ELSO) and American Heart Association (AHA) guidelines now recognize ECPR as a technique that can be considered in select cardiac arrest patients. Refractory cardiac arrest is defined by the lack of return of spontaneous circulation within a period of at least 30 min of high-quality cardiopulmonary resuscitation (CPR). Two main factors impact the outcomes in these cases and the survival with ECPR (Extracorporeal Cardiopulmonary Resuscitation): The cause of arrest and the quality of resuscitation. Better outcomes have been seen when ECPR is implemented within the first 30 min of arrest [4]. ECPR in pediatric patients has been recommended in children with heart disease when the etiology of the arrest is thought to be “amenable to recovery or transplantation”, according to The American Heart Association (AHA) [5].

A 2016 evaluation of the AHA’s get with the Guidelines–Resuscitation registry revealed that for children with in-hospital CPR > 10 min duration, ECPR has been associated with improved survival to hospital discharge and survival with favorable neurologic outcomes [6]. Despite increased experience with ECMO in adults and children, ideal criteria for pediatric ECPR have not been completely described. This is in part to different factors that affect the outcomes and variations in resources and management strategies in hospitals, for example the availability of resources in different institutions, and the lack of a clear protocol within the institutions to implement these strategies. For that reason, a methodical approach to ECPR implementation and monitoring of long-term outcomes is essential.

Patients with congenital heart disease who are taken to surgery have a higher risk of presenting neurological lesions in the postoperative period; this risk is higher in procedures using cardiopulmonary bypass [7]. The actual incidence of neurological complications is difficult to establish and varies in the literature. In neonatal and infant patients, it is difficult to assess neurological compromise due to limitations in functional assessment because of age and apparently due to potential brain plasticity that may only become evident in very long-term follow-up [8, 9].

The pediatric population appears to be more vulnerable to postoperative cardiac dysfunction when they undergo definitive repair of congenital heart disease [10]. The abnormal elevation of myocardial enzymes is associated with worse outcomes in the early and late postoperative periods. This includes the longer time of ventilatory support, prolonged stay in the ICU, and even mortality [11, 12]. Tan et al., in a meta-analysis of 9 randomized controlled clinical trials involving 793 patients under 18 years of age undergoing Cardiac Surgery, describe the cardioprotective effect of ischemic preconditioning during the early postoperative period. Troponin I levels at 6 h and inotropic scores at 4–6 h were lower, as were the ventilatory support and the ICU stay [13].

Higher lactate levels, higher creatinine levels, and prolonged ECMO duration were associated with higher mortality. Early diagnosis and intervention of residual anatomical problems could improve survival. Bleeding and renal failure were the most common complications and the incidence of renal failure may be correlated with longer hypoperfusion duration [1]. These findings are similar to those reported in a cohort of 67 patients who underwent E-CPR, where a 33.8% survival to hospital discharge was described (24 of 67 patients). The most frequent complications, reported in the literature, were bleeding (26 of 67), kidney failure (19 of 67), and neurological injury (14 of 67). Although the outcomes are not unfavorable, the complication rates were high, and of these complications, renal failure is associated with high mortality [14].

## Conclusion

Although the criteria for the use of E-CPR in the early postoperative period for correction of congenital heart disease are not well described in the literature, time (<30 min) is considered one of the most important long-term neurological outcome factors. The case of ECPR with CPR time > 30 min and a good neurological outcome is reported here, suggesting that factors such as neuroplasticity and ischemic preconditioning could be associated with improved outcomes over time.

Reviewing the institutional experience of high-volume pediatric ECMO centers takes an initial step toward process improvement and more favorable outcomes. While our data adds to the growing literature supporting the use of pediatric ECPR, larger, multicenter studies are required to meaningfully describe the neurologic outcome and functional status of this population; survival is an essential but insufficient measure [6].

## Data Availability

The research data are available on request from the authors.
